# Trunk motion and gait characteristics of pregnant women when walking: report of a longitudinal study with a control group

**DOI:** 10.1186/1471-2393-13-71

**Published:** 2013-03-20

**Authors:** Wendy L Gilleard

**Affiliations:** 1 School of Health and Human Sciences, Southern Cross University, PO Box 157, Lismore, NSW, 2480, Australia

**Keywords:** Kinematics, Pelvis, Post-birth, Pregnancy, Step width, Stride length, Temporospatial, Thorax, Trunk, Walking

## Abstract

**Background:**

A longitudinal repeated measures design over pregnancy and post-birth, with a control group would provide insight into the mechanical adaptations of the body under conditions of changing load during a common female human lifespan condition, while minimizing the influences of inter human differences. The objective was to investigate systematic changes in the range of motion for the pelvic and thoracic segments of the spine, the motion between these segments (thoracolumbar spine) and temporospatial characteristics of step width, stride length and velocity during walking as pregnancy progresses and post-birth.

**Methods:**

Nine pregnant women were investigated when walking along a walkway at a self-selected velocity using an 8 camera motion analysis system on four occasions throughout pregnancy and once post birth. A control group of twelve non-pregnant nulliparous women were tested on three occasions over the same time period. The existence of linear trends for change was investigated.

**Results:**

As pregnancy progresses there was a significant linear trend for increase in step width (*p* = 0.05) and a significant linear trend for decrease in stride length (*p* = 0.05). Concurrently there was a significant linear trend for decrease in the range of motion of the pelvic segment (*p* = 0.03) and thoracolumbar spine (*p* = 0.01) about a vertical axis (side to side rotation), and the pelvic segment (*p* = 0.04) range of motion around an anterio-posterior axis (side tilt). Post-birth, step width readapted whereas pelvic (*p* = 0.02) and thoracic *(p* < 0.001) segment flexion-extension range of motion decreased and increased respectively. The magnitude of all changes was greater than that accounted for with natural variability with re testing.

**Conclusions:**

As pregnancy progressed and post-birth there were significant linear trends seen in biomechanical changes when walking at a self-determined natural speed that were greater than that accounted for by natural variability with repeated testing. Not all adaptations were resolved by eight weeks post birth.

## Background

Walking is an essential daily activity and important in controlling adipose tissue weight gain associated with pregnancy [1]. The mechanics of walking however may be affected as pregnancy is characterized by maternal changes in shape and dimensions, particularly in the trunk. As pregnancy progresses, the lower trunk segment inertial characteristics show a significantly larger rate of increase than any other body segments [2]. With rapid changes in mass, and moment of inertia [2], trunk segment kinematics may be altered in daily activities such as walking. The possibility of altered kinematics is important as this may also affect the kinetics and hence musculoskeletal demands on the trunk segments.

Much of the focus on trunk mechanical adaptations in pregnancy has been on static postures [3-9]. There are few reports of trunk segment motion during pregnancy when walking. Foti et al. [10] reported increased peak anterior pelvic tilt in late pregnancy when compared to one year post-birth with no differences in pelvic rotation in the transverse or coronal plane. No control group was used and differences may have been due to natural human variability with retesting. Wu et al. [11] found that for pregnant women the range of motion amplitudes in the transverse plane of the pelvis and thoracic segments, and thoracolumbar spine were similar to a control nulliparous group, however the intra-individual standard deviations were significantly smaller. Wu et al. [11], however, included women between 20 and 34 weeks gestation in a single group for which there would have been wide variation in the lower trunk segment inertias. The within group variability may have precluded finding a significant difference in amplitude although a consistent reduction in comparison to the control group was noted [11].

Temporospatial gait characteristics such as velocity [12], stride length [12] and step width [13] effect, or are affected by, trunk segment kinematics during walking [12,13]. Therefore it is essential, in order to more fully understand any potential kinematic effects of pregnancy, to include these temporospatial gait characteristics in the investigation as they may also influence the parameters. While several studies have investigated temporospatial characteristics of gait in late pregnancy [11,14-17] the results are equivocal. There is also a paucity of reports investigating changes in these parameters as pregnancy progresses.

While understanding of the effects of pregnancy on trunk segment motion during walking and temporospatial characteristics has increased using cross sectional or comparison to post-birth designs, further information is required about adaptations as pregnancy progresses and in the early post-birth period. A longitudinal repeated measures design over pregnancy and post-birth, with a control group would provide insight into the mechanical adaptations of the body under conditions of changing load during a common female human lifespan condition, while minimizing the influences of inter human differences. As pregnancy is characterized by continuous changes over time, changes may be expected to show systematic trends as the pregnancy progresses. The aim of this study was to investigate the linear trends for change in the range of motion of the thoracic and pelvic segments and thoracolumbar spine, and the temporospatial characteristics for walking at a self-determined natural speed as pregnancy progressed and in the early post birth period using a longitudinal retest design. Comparisons were also made with the typical range and natural variability from test to retest established using nulliparous subjects. It was hypothesized that range of motion of the thoracic and pelvic segments and thoracolumbar spine, and the temporospatial characteristics would alter as pregnancy progressed with a re-adaptation post-birth.

## Methods

A volunteer sample of convenience consisting of nine maternal subjects (mean age 32.6(4.3) years, height 163.7(6.6) cm, 38 weeks gestation mass 76.8(10.9) kg (n = 8), post birth mass 66.8(10.3) kg) and twelve nulliparous subjects (mean age 28.9(4.1) years, height 165.4(4.9) cm, mass at third test 62.2(7.4) kg) volunteered and were included in the study which was approved by The University of Sydney Ethics Committee. Maternal subjects included five primigravidas and four multigravidas. The maternal group was tested at 18 weeks or less, 24 weeks, 32 weeks and 38 weeks gestation and again at eight weeks post-birth (Maternal Sessions 1 to 5). The control group was tested initially then re-tested 16 weeks (Control Session 2) and 32 weeks later (Control Session 3). The first test session for each group was considered to be a familiarization session and therefore the data was not included in further analysis.

**Figure 1:**
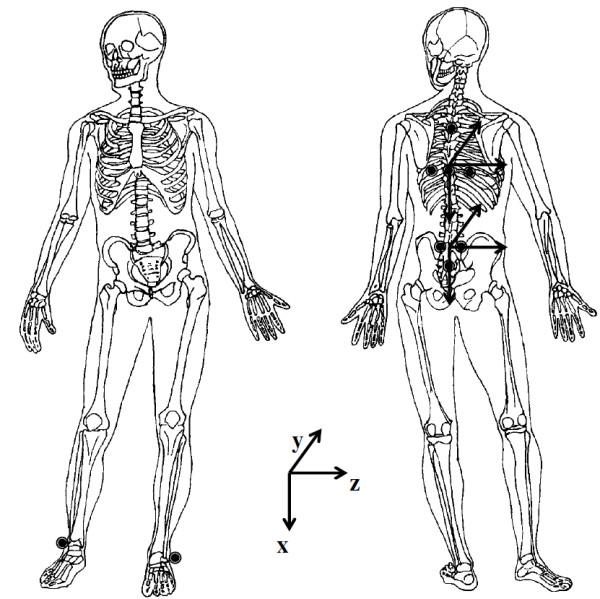
Marker placement in anterior and posterior views with segment coordinate system.

A Motion Analysis Corporation™ Expert Vision System™ ^a^ together with eight synchronized cameras (NEC T1-23A), was used to record one complete gait cycle at 60 Hz. Prior to each test session the filming space was calibrated, with the calibration frame enclosing a space 0.8 m wide, 2 m high and 1.2 m long. The location of the calibration frame was approximately central to the camera positions, over the force plate and all movements were performed within or immediately adjacent to the space described by the location of the calibration frame. The beginning of the gait cycle was determined by right heel strike on an embedded Kistler™ 9281 force platform ^b^ (sampling at 960Hz) placed along the plane of progression. EVa HiRes™ version 4.0 (Motion Analysis Corporation™) was used to identify the three dimensional trajectories of the markers. Light weight, 2 cm diameter, spherical retro-reflective markers, were used to define body segments similar to Crosbie et al. [18] as shown in Figure 1. The thoracic segment was defined by markers on T_4_ and T_8_ spinous processes, and left and right angle of 8th rib. The pelvic segment consisted of markers on both posterior superior iliac spines, and the S_4_ spinous process. Markers were also placed on the left and right lateral ankle malleoli.

Data collection at each session included a reference position and walking trials. For the reference position, subjects were required to stand within the previously calibrated space in an erect posture, arms hanging naturally by the side of the body and looking straight ahead at an eye level fixed point. Foot position in the reference trial was standardized by standing on the force platform such that the lateral borders of the feet were aligned in a parallel manner with the edges of the force platform. Subjects walked at a self-determined natural speed across a laboratory approximately 20 meters long for three trials where the subjects naturally and consistently contacted the force platform (located centrally to the walkway) at right heel strike while looking at an eye level fixed point throughout the trial.

Motion data were processed using Kintrak™ version 5.7^*^ with a cut off frequency of 6Hz (4th order zero phase shift Butterworth filter). Angular rotations were determined using segmental and joint coordinate systems. The coordinate system for each segment was defined such that its axes originated from the determined joint centre. The segment coordinate system was defined with positive y in the direction of forward travel, negative x was vertically up and positive z followed the right hand rule to the right. Using an approach similar to Crosbie et al. [18], for the thoracic segment the joint centre was located midway between the left and right 8th rib angles and aligned vertically and horizontally in line with the angle of the right 8th rib (Figure 1). For the pelvis the joint centre was located midway between the left and right Posterior Superior Iliac Spines, aligned vertically with the right Posterior Superior Iliac Spine and aligned horizontally in line with the right Posterior Superior Iliac Spine adjusted by 62.5 mm (Figure 1).

A matrix was then determined from information on the location of at least three marker points in the segment coordinate system and the location of the same marker points within the laboratory coordinate system. The matrix served to represent the orientation of the segment coordinate system with respect to the laboratory coordinate system. The set of a minimum of three points for each segment was assumed to represent a rigid body. As such, an embedded coordinate system was defined, and its relationship to the segment during dynamic motion remained unchanged.

For the pelvic and thoracic segments, angular motion of each segment in space was calculated using the segment coordinate system with respect to the laboratory coordinate system. The reference position was used to represent zero angular displacement of the pelvic and thoracic segments. To investigate the relative rotational patterns of the pelvic and thoracic segments (thoracolumbar spine), the conventions used for calculation of joint angle using the segment coordinate system were applied where, two segment coordinate system axes were assumed to be embedded in each adjacent segment. The embedded axis in the thoracic segment served as the mediolateral axis and the one in the pelvis served as the longitudinal. The fixed axes moved with the adjacent segments such that the spatial relationship between them changed with motion. The third axis was mutually perpendicular to the two body fixed axes.

Stride length and step width were determined from the spatial trajectories of the foot markers, and the force platform data.

### Data analysis

A repeated measures ANOVA with planned contrasts and a Bonferroni adjustment for multiple contrasts was used to examine each cluster of variables. The temporospatial cluster included velocity, stride length, and step width. The trunk kinematic cluster included range of motion for the thoracic and pelvic segments and the thoracolumbar spine in the sagittal, coronal and transverse planes. As pregnancy is characterized by continuous changes over time, and may be expected to show systematic trends as the pregnancy progresses, a polynomial planned contrast was used to investigate the existence of linear trends between 24 to 38 weeks gestation. Simple planned contrasts were used to investigate differences between 38 weeks gestation and 8 weeks post-birth. It is also possible that changes attributed to pregnancy were actually related to variations in human motion which occur naturally over time or were due to the psychosocial effects of repeated testing. Therefore for any significant linear trend, the magnitude of the change by the maternal subjects was also compared with standard error of the measurement (SEM) associated with retesting established from the control group between Control Session 2 and Control Session 3. For changes less than the natural variability of the control group, a note was made against the significant trend in the data tables.

Missing data for one maternal subject occurred due to early delivery at 38 weeks gestation. Linear extrapolation on the remaining three pregnancy test session data points was used to predict missing data. These data represented 2.8% of the total maternal data set for each variable.

## Results

The temporospatial parameter results are summarized in Tables 1 and 2. As pregnancy progressed there was a significant decreasing linear trend in stride length (F_linear_ = 5.52, *p* = 0.05) and a significant increasing linear trend in step width (F_linear_ = 5.54, *p* = 0.05). The mean changes over pregnancy were greater than the natural variability associated with retesting as indicated by the SEM (Table 2). There were no significant linear trends for change as pregnancy progressed in walking velocity. Post-birth, step width (F = 8.54, *p* = 0.02) was smaller than in late pregnancy.

**Table 1:** Maternal temporospatial variables during pregnancy including ANOVA F values for linear trends

	**Maternal** **24 weeks**	**32 weeks**	**38 weeks**	**F**_**linear**_	**P**
Velocity (ms^-1^)	1.3 (0.1)	1.3 (0.1)	1.3 (0.1)	1.20	0.32
Stride length (cm)	141.1 (12.5)	141.1 (11.5)	138.1 (12.3)	5.52	0.05*
Step width (cm)	19.6 (3.5)	20.1 (4.6)	20.8 (4.1)	5.54	0.05*

*significant linear trend at p ≤ 0.05.

**Table 2:** maternal post-birth, and control temporospatial variables and standard error of the measurement

	**Maternal** **Post-Birth**	**F**	**P**	**Control** **Session 3**	**SEM**
Velocity (ms^-1^)	1.3 (0.1)	0.56	0.48	1.3 (0.1)	± 0.05
Stride length (cm)	139.5 (10.4)	1.45	0.26	136.4 (11.0)	± 2.00
Step width (cm)	19.1 (4.3)	8.54	0.02^+^	18.8 (3.4)	± 1.15

F and P values for difference between 38 weeks gestation and post-birth.

^+^ significant difference between 38 weeks and Post-Birth at p ≤ 0.05.

**Figure 2:**
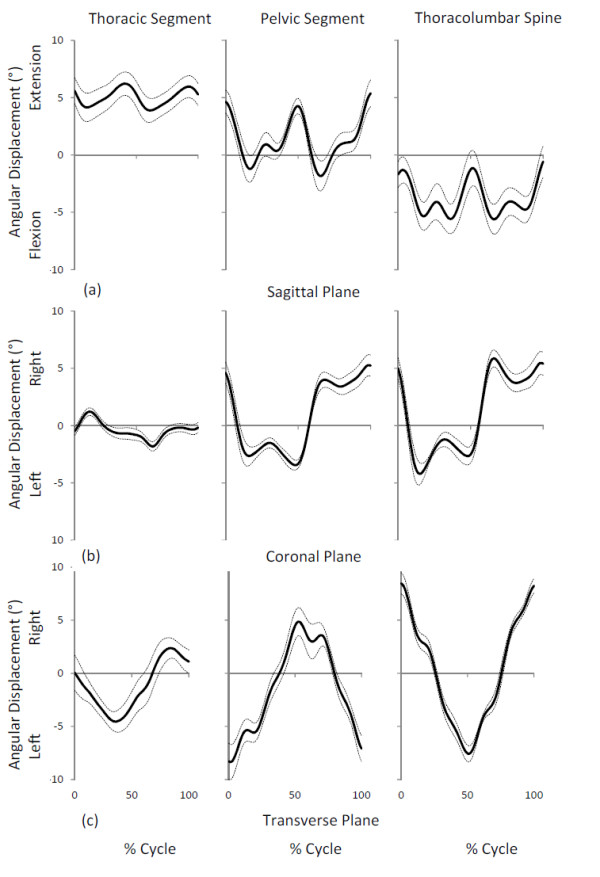
**Maternal group patterns of motion at 38 weeks gestation.** Mean ± Standard Error of Mean of thoracic segment, pelvic segment and thoracolumbar spine displacement: **(a)** sagittal plane where positive values indicates thoracic segment, pelvic segment and thoracolumbar spine extension, **(b)** coronal plane where positive values indicates thoracic segment, pelvic segment and thoracolumbar spine side flexion to the right, and **(c)** transverse plane where positive values indicates thoracic segment, pelvic segment and thoracolumbar spine axial rotation to the right. 0% of movement right heel strike.

The patterns of motion for the thoracic and pelvic segments and thoracolumbar spine are shown in Figure 2. The range of motion results for the thoracic and pelvic segments and thoracolumbar spine are summarized in Tables 3 and 4. The transverse plane range of motion of the pelvic segment (F_linear_ = 6.53, *p* = 0.03) and thoracolumbar spine (F_linear_ = 11.66, *p* = 0.01) showed a significant decreasing linear trend as pregnancy progressed. The mean changes during pregnancy were greater than the natural variability associated with retesting as indicated by the SEM, and there was no significant reversal of this trend by eight weeks post-birth (Tables 3 and 4).

**Table 3:** Range of motion of the trunk segments during pregnancy including ANOVA F values for linear trends

**Plane**	**Maternal** **24 weeks**	**32 weeks**	**38 weeks**	**F**_**linear**_	**P**
Transverse					
Thorax (°)	9.8 (3.0)	10.7 (3.7)	10.3 (2.5)	0.22	0.66
Pelvis (°)	16.8 (5.9)	15.9 (7.4)	13.9 (6.7)	6.54	0.03*
Thoracolumbar (°)	22.0 (3.8)	18.5 (2.6)	17.3 (3.9)	11.66	0.01*
Coronal					
Thorax (°)	3.8 (1.2)	4.15 (1.2)	4.16 (1.2)	0.36	0.57
Pelvis (°)	14.6 (3.9)	10.59 (2.2)	11.03 (3.2)	6.41	0.04*
Thoracolumbar (°)	15.4 (3.3)	12.80 (2.7)	12.54 (2.3)	4.39	0.07
Sagittal					
Thorax (°)	4.1 (0.9)	3.9 (0.9)	3.3 (0.8)	4.69	0.06
Pelvis (°)	9.9 (3.9)	8.9 (3.1)	9.0 (3.1)	0.26	0.62
Thoracolumbar (°)	9.0 (2.6)	7.4 (2.7)	6.9 (2.7)	4.23	0.07

* significant linear trend at p ≤ 0.05.

**Table 4:** Maternal Session post-birth and control range of motion of the trunk segments and standard error of the measurement

**Plane**	**Maternal** **Post-Birth**	**F**	**P**	**Control** **Session 3**	**SEM**
Transverse					
Thorax (°)	10.8 (3.3)	0.10	0.76	7.9 (1.8)	±1.4
Pelvis (°)	14.3 (5.7)	0.14	0.72	13.4 (4.2)	±2.3
Thoracolumbar (°)	20.5 (5.9)	3.44	0.10	18.5 (3.7)	±2.9
Coronal					
Thorax (°)	4.4 (1.4)	0.09	0.77	4.3 (1.2)	±0.7
Pelvis (°)	13.0 (4.1)	1.71	0.23	10.7 (2.4)	±2.1
Thoracolumbar (°)	14.9 (3.7)	2.17	0.18	13.68 (3.3)	±1.7
Sagittal					
Thorax (°)	4.8 (1.0)	61.65	<0.001^+^	4.4 (1.7)	±1.0
Pelvis (°)	6.8 (1.3)	8.62	0.02^+^	8.6 (4.2)	±1.7
Thoracolumbar (°)	7.2 (2.3)	0.07	0.80	8.6 (2.8)	±1.67

F and P values are for difference between 38 weeks gestation and post-birth.

^+^ significant difference between 38 weeks and Post-Birth at p ≤ 0.05.

The pelvic segment in the coronal plane showed a significant linear trend for decreased range of motion (F_linear_ = 6.41, *p* = 0.04) with advancing pregnancy with the mean changes greater than the natural variability associated with retesting (Tables 3 and 4). There was no significant reversal of this trend by eight weeks post-birth (Table 4). Sagittal plane range of motion for the thoracic and pelvic segment and the thoracolumbar spine, showed no significant linear trends with advancing pregnancy. Post-birth, however, the thoracic segment range of motion was larger (F = 61.65, *p* < 0.001) and the pelvic segment range of motion was smaller (F = 8.62, *p* = 0.02) in comparison to late pregnancy. These changes in means were greater than the natural variability associated with retesting as indicated by the SEM (Table 4).

## Discussion

The study aimed to investigate the linear trends for change in the range of motion of the thoracic and pelvic segments and thoracolumbar spine, and the temporospatial characteristics of velocity, stride length and step width for walking at a self-determined natural speed as pregnancy progressed and in the early post birth period using a longitudinal retest design. Comparisons were also made with the typical range and natural variability from test to retest established using nulliparous subjects. The patterns of motion, range of motion and inter-subject variability for the Control group session 3 were similar to previous reports [19,20]. Direct comparisons between the literature are problematic due to differences resulting from different modeling approaches for the trunk segments [21] and differences in gait between genders [20]. As a longitudinal retest design may be affected by variability in human motion which occur naturally over time or were due to the psychosocial effects of repeated testing it was important to establish the normal variability over time. The SEM was a relatively small proportion of the mean and within the inter-subject variability as indicated by the Standard Deviation.

As pregnancy progressed there were biomechanical changes when walking greater than that accounted for by natural variability with retesting. Step width increased as pregnancy progressed similar to Bird et al. [14] and returned to non-pregnant values by eight weeks post-birth in agreement with Lymbery and Gilleard [16]. It has been argued that increased step width reflects a need for augmented stability during pregnancy [14], however Foti et al. [10] suggested that increased step width is a consequence of increased pelvic width. Whether the increased step width is a mechanical consequence of increased pelvic width or a response to a need for stability is unresolved.

Regardless of the underlying explanation for an increased step width, a consequence of increased step width is that the foot is more lateral with each step. The lateral displacement of the body is therefore increased with each step as the body is shifted over the weight bearing leg. Hence the increased body side to side motion which is often described anecdotally as “waddling“gait can be observed. The increased hip abduction moment and power in stance at late pregnancy reported by Foti et al. [10] may reflect increased muscle activity to required move the larger trunk mass over the more lateral supporting leg. A secondary consequence of this may also be the reduced pelvic segment coronal plane motion seen in the present study as the higher abduction muscle activity may reduce the pelvic drop on the non-supported side.

Decreasing the stride length results in reduced magnitude of pelvic segment and thoracolumbar spine rotation in the transverse plane in healthy adults when walking [12]. Therefore the decrease in stride length as pregnancy progressed may have the cause of the reduced range of motion for the pelvic segment and thoracolumbar spine in the transverse plan in the present study and also reported by Wu et al. [11]. However it is also possible the converse is true and that the reduced range of trunk segment motion in the transverse plane may have resulted in a reduced stride length. The magnitude of the change in range of motion is small and may be related to a lower trunk moment of inertia increase [2] as pregnancy progresses. As the moment of inertia is increased, control of angular momentum in the transverse and coronal planes may be achieved by reducing the pelvic segment range of motion by decreasing stride length. A reduced range of motion may also reflect a requirement for higher level of muscular activity as pregnancy progresses, as restricting excessive trunk motion is a function of the lumbar Erector Spinae muscles [22]. Further studies are warranted to examine any changes in posterior trunk muscle activity during walking as pregnancy progresses.

There was no change in sagittal plane kinematics as pregnancy progressed, however, the relatively large variances (Table 3) may indicate pregnant women have an individual dynamic response to the increased inertial effects of pregnancy as suggested for trunk static posture [3,7,9] . Post-birth the altered range of motion in the pelvic and thoracic segments may be related to the functional capability of anterior abdominal wall musculature and the posterior trunk muscles at this time. Stabilization of the pelvis by the abdominal muscles is compromised up to eight weeks post-birth [23] and the fatigability of the trunk extensors is decreased in that period [24]. Thus an imbalance between the anterior and posterior postural muscles may exist post-birth, and this may be reflected in the decreased pelvic segment range of motion. The increased range of motion of the thoracic segment may reflect a counter motion with the net effect of no change in the thoracolumbar spine range of motion. Further studies are warranted to examine any changes in anterior and posterior trunk muscle activity during walking as pregnancy progresses.

Velocity showed no consistent change as pregnancy progressed similar to the longitudinal study by Golomer et al. [15]. The result was in contrast to previous reports [11,16,25]. A familiarization session was not used in these previous studies so it is possible that the reported decreased velocity reflects novel test conditions for pregnant women who are also aware of difficulties in everyday tasks [26]. The velocity at late pregnancy in this study was similar to that reported by McCrory et al. [25], however the control group velocity was slower. The present study also shows that velocity varies with repeated data collections at 0.1 ms^-1^ SEM (Table 2). Therefore regular testing over a pregnancy is required to demonstrate if reported changes in velocity have a consistent direction over the term of a pregnancy.

The study is limited by the small number of participants which has precluded the use of simple planned comparison as pregnancy progresses. The results of the study do confirm linear trends for change in some dependent variables. Therefore further studies are recommended with larger numbers to edify the magnitude of such changes. The study was unable to identify the causes of the observed changes in thoracic and pelvic segment motion. It is possible that the identified changes are the result of changes in muscle activity. Therefore further studies are required to examine anterior and posterior trunk muscle activity during gait as pregnancy progresses.

## Conclusions

As pregnancy progressed and post-birth there were biomechanical changes for walking at a self-determined natural speed greater than that accounted for by natural variability with repeated testing. As pregnancy progressed there was a significant decreasing linear trend in stride length, a significant increasing linear trend in step width with no significant linear trend seen for velocity. Post birth the step width readapted to returned to normal.

The transverse plane range of motion of the pelvic segment and thoracolumbar spine showed a significant decreasing linear trend as pregnancy progressed with no significant reversal of this trend by eight weeks post-birth. The pelvic segment in the coronal plane showed a significant linear trend for decreased range of motion with advancing pregnancy with no significant reversal of this trend by eight weeks post-birth. Sagittal plane range of motion for the thoracic and pelvic segment and the thoracolumbar spine, showed no significant linear trends with advancing pregnancy. Post-birth, however, the thoracic segment range of motion was larger and the pelvic segment range of motion was smaller in comparison to late pregnancy.

## Endnotes

^a^Motion Analysis Corp., 3617 West wind Boulevard, Santa Rosa, California 95403

^b^Kistler, Winterthur, Switzerland

## Competing interests

The author declares that she has no competing interests.

## Pre-publication history

The pre-publication history for this paper can be accessed here:

http://www.biomedcentral.com/1471-2393/13/71/prepub
